# Niemann-Pick Type C2 Protein Regulates Free Cholesterol Accumulation and Influences Hepatic Stellate Cell Proliferation and Mitochondrial Respiration Function

**DOI:** 10.3390/ijms19061678

**Published:** 2018-06-05

**Authors:** Yuan-Hsi Wang, Yuh-Ching Twu, Chung-Kwe Wang, Fu-Zhen Lin, Chun-Ya Lee, Yi-Jen Liao

**Affiliations:** 1 School of Medical Laboratory Science and Biotechnology, College of Medical Science and Technology, Taipei Medical University, Taipei 110, Taiwan; m609105001@tmu.edu.tw (Y.-H.W.); linfuzhen4589@gmail.com (F.-Z.L.); s9150921@hotmail.com (C.-Y.L.); 2 Department of Biotechnology and Laboratory Science in Medicine, National Yang-Ming University, Taipei 112, Taiwan; yctwu@ym.edu.tw; 3 Department of International Medicine, Taipei City Hospital Ranai Branch, Taipei 106, Taiwan; ckwang88@ms74.hinet.net

**Keywords:** Niemann-Pick type C2, hepatic stellate cells, free cholesterol, platelet-derived growth factor BB, mitochondrial function

## Abstract

Liver fibrosis is the first step toward the progression to cirrhosis, portal hypertension, and hepatocellular carcinoma. A high-cholesterol diet is associated with liver fibrosis via the accumulation of free cholesterol in hepatic stellate cells (HSCs). Niemann-Pick type C2 (NPC2) plays an important role in the regulation of intracellular free cholesterol homeostasis via direct binding with free cholesterol. Previously, we reported that NPC2 was downregulated in liver cirrhosis tissues. Loss of NPC2 enhanced the accumulation of free cholesterol in HSCs and made them more susceptible to transforming growth factor (TGF)-β1. In this study, we showed that knockdown of NPC2 resulted in marked increases in platelet-derived growth factor BB (PDGF-BB)-induced HSC proliferation through enhanced extracellular signal-regulated kinases (ERK), p38, c-Jun N-terminal kinases (JNK), and protein kinase B (AKT) phosphorylation. In contrast, NPC2 overexpression decreased PDGF-BB-induced cell proliferation by inhibiting p38, JNK, and AKT phosphorylation. Although NPC2 expression did not affect caspase-related apoptosis, the autophagy marker light chain 3β (LC3B) was decreased in NPC2 knockdown, and free cholesterol accumulated in the HSCs. The mitochondrial respiration functions (such as oxygen consumption rate, ATP production, and maximal respiratory capacity) were decreased in NPC2 knockdown, and free cholesterol accumulated in the HSCs, while NPC2-overexpressed cells remained normal. In addition, NPC2 expression did not affect the susceptibility of HSCs to lipopolysaccharides (LPS), and U18666A treatment induced free cholesterol accumulation, which enhanced LPS-induced Toll-like receptor 4 (TLR4), nuclear factor kappa-light-chain-enhancer of activated B cells (NF-κB) p65 phosphorylation, interleukin (IL)-1 and IL-6 expression. Our study demonstrated that NPC2-mediated free cholesterol homeostasis controls HSC proliferation and mitochondrial function.

## 1. Introduction

Liver fibrosis is a response to chronic liver injury due to various factors such as viral infection (hepatitis B and hepatitis C), alcohol consumption, non-alcoholic steatohepatitis (NASH), non-alcoholic fatty liver disease, autoimmune-disorder-induced hepatitis, and cholestatic liver diseases [1]. Regardless of the underlying cause, persistent liver injury generates chronic inflammation and results in an abnormal wound healing response to mitigate the damage [1]. The fibrotic response in the liver leads to an excessive accumulation of extracellular matrix (ECM) components [2]. Once the liver fibrosis progresses over time, the scar tissue disrupts the architecture of the liver and results in the development of cirrhosis and liver failure [1,2]. Hepatic stellate cells (HSCs) are the major cell type responsible for extracellular matrix deposition and fibrosis formation in the liver. HSCs are located in the Disse space within the liver sinusoid [3]. In a quiescent state, HSCs are the main cells for vitamin A storage and retinoic acid homeostasis regulation [4]. Upon stimulation, quiescent HSCs are activated by inflammatory mediators and undergo a process of transdifferentiation into fribrogenic myofibroblast-like cells [4,5]. Activated HSCs increase the expression of α-smooth muscle actin (α-SMA) and the production of excessive collagen, thereby accelerating the progression of liver fibrosis [4]. Myofibroblast-like cells in the injured liver also contribute to liver cancer progression and metastasis [6,7].

Among the cellular mechanisms involved in liver fibrogenesis, platelet-derived growth factor (PDGF) plays a significant role in the activation of HSCs [8]. PDGF-BB is the most effective stimulator in HSC proliferation and intracellular signaling. When PDGF recognizes receptors on the HSCs, it will induce the dimerization of β-PDGF receptor subunits and lead to auto-phosphorylation [9]. This process initiates the downstream phosphorylation of mitogen-activated protein kinases (MAPK/ERK) and protein kinase B (AKT) of the phosphoinositide-3-kinases (PI3K) pathways, and triggers HSC proliferation [8]. A previous study has shown that liver tissue obtained from chronic liver diseases expressed PDGF and its receptor subunits, which were especially correlated with the degree of liver fibrosis [10]. 

Increasing evidence indicates that the lipopolysaccharide (LPS)/Toll-like receptor 4 (TLR4) signaling pathway in HSCs is associated with liver fibrogenesis [11]. LPS comes from the outer membrane of Gram-negative bacteria, and the increasing permeability of the gut in chronic liver disease patients allows the translocation of LPS into the liver via the portal vein [12]. Previous research has shown that activated HSCs rather than Kupffer cells are the primary target for TLR4 ligands, which enhance liver fibrosis [13,14]. Upon LPS binding the receptor, TLR4 subsequently activates a downstream-signaling cascade such as transcription factor NF-κB (p65), which in turn regulates the expression of pro-inflammatory cytokines and controls cell survival [15].

In humans, a higher dietary consumption of cholesterol is associated with a higher risk of cirrhosis or liver cancer in both unadjusted and adjusted analyses [16]. Several studies have shown that a high-cholesterol diet exacerbates carbon tetrachloride (CCl_4_) and bile duct ligation induced liver fibrosis in rodents via the accumulation of free cholesterol (but not cholesterol ester) in HSCs [17]. The free cholesterol accumulation increases the sensitization of HSCs to TGF-β and plays an important role in the pathogenesis of liver fibrosis [17,18]. Encoding from the Niemann-Pick Type C2 (*NPC2*) gene, NPC2 protein is a free cholesterol-binding protein that mainly resides within endosomes and lysosomes compartments [19,20]. Most NPC2 deficient patients die from neurodegenerative diseases, some patients also have liver failure [21,22]. Previously, we reported that NPC2 expression was downregulated in human liver cirrhosis and hepatoma tissues [23]. Recently, we found that the loss of NPC2 expression in HSCs results in higher susceptibility to TGF-β1 treatment [24]. However, the molecular mechanisms of NPC2 expression in the HSCs towards PDGF-BB-induced proliferation, the LPS-induced inflammatory reaction, and mitochondrial function have not been explored in detail.

In this study, we provided new insights into the mechanisms linking NPC2 deficiency-induced free cholesterol accumulation and influenced HSCs proliferation and mitochondrial function. Regulation of NPC2 in HSCs may therefore represent a new treatment strategy for liver fibrosis. 

## 2. Results

### 2.1. NPC2 Downregulation and Free Cholesterol Accumulation Enhanced PDGF-BB-Induced Proliferation in HSCs

**Figure 1:**
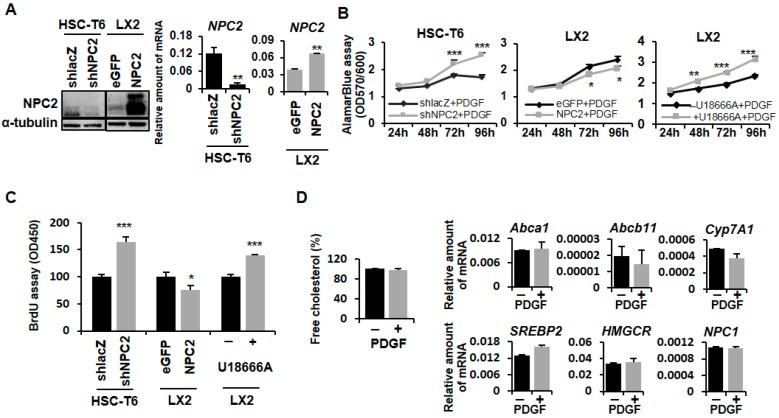
NPC2 downregulation induced free cholesterol accumulation which influenced PDGF-BB-induced cell proliferation in HSCs. (**A**) Western blot and real-time PCR were used to assess *NPC2* gene expression in HSC-T6 shNPC2 and LX2 NPC2 stable cells; (**B**) Different lentiviruses infected HSC-T6 (shlacZ and shNPC2), LX2 (eGFP and NPC2) stable cells, and 1 µM U18666A pretreated LX2 cells were treated with 10 ng/mL PDGF-BB for the indicated time periods, and the cell viability was analyzed by adding 10 μL alamarBlue^®^ reagent for 2.5 h and evaluating the absorbance at 570/600 nm; (**C**) Different lentiviruses infected HSC-T6 (shlacZ and shNPC2), LX2 (eGFP and NPC2) stable cells, and 1 µM U18666A pretreated LX2 cells were treated with 10 ng/mL PDGF-BB for 72 h, and the cell viability was analyzed by using the BrdU cell proliferation assay and evaluating the absorbance at 450 nm; (**D**) LX2 cells were treated with or without 10 ng/mL PDGF-BB overnight and then subjected to intracellular free cholesterol quantification. Real-time PCR was used to assess *Abca1*, *Abcb11*, *Cyp7A1*, sterol regulatory element-binding proteins *(SREBP2*), 3-hydroxy-3-methyl-glutaryl-coenzyme A reductase (*HMGCR*), and *NPC1* in HSC-T6 cells. Data are shown as mean ± SD. * *p* < 0.05; ** *p* < 0.01; *** *p* < 0.001 vs. black line/bar. Each experiment was performed using three independent replicates with similar results.

In our previous research, we established *NPC2* gene overexpression and knockdown HSCs cell lines [24]. Compared to the shlacZ control, HSC-T6 shNPC2 cells showed decreased NPC2 expression in Western blot and real-time PCR experiments. On the other hand, LX2 NPC2 cells had higher NPC2 expression than LX2 enhanced green fluorescent protein (eGFP) cells in both protein and mRNA levels (Figure 1A). The free cholesterol levels in the HSC-T6 shNPC2 and LX2 NPC2 groups were analyzed in our previous research, where the level of free cholesterol was significantly higher in the NPC2 knockdown cells than in the shlacZ control cells. However, no difference was noted between the eGFP control and NPC2-overexpressed cells [24]. 

PDGF-BB is a strong HSC mitogen which can promote HSC proliferation and activation [25]. To study whether NPC2 is involved in the proliferation process of HSCs, we used the alamarBlue^®^ cell viability assay and BrdU assay to investigate the PDGF-BB-induced HSC proliferation. Compared to the shlacZ control, HSC-T6 shNPC2 cells had a greater proliferative capacity (Figure 1B,C, left). In contrast, the overexpression of NPC2 in the LX2 cells had slightly suppressed cell proliferation under the PDGF-BB treatment (Figure 1B,C, middle). Since the function of the NPC2 protein is to control intracellular free cholesterol homeostasis [19,20], we next determined whether direct free cholesterol accumulation in HSCs would cause the same response toward PDGF-BB treatment like the NPC2 knockdown HSCs. U18666A, an inhibitor of free cholesterol transport, is a positive control to induce free cholesterol accumulation [26,27,28,29]. As shown in Figure 1B,C, on the right, similar results were observed where U18666A-treated HSCs had a significant increase in cell proliferation when compared with the control, while PDGF-BB treatment did not influence the free cholesterol levels in HSCs (Figure 1D, left). The expression of cholesterol regulation related genes *Abca1*, *Abcb11*, *Cyp7A1*, *SREBP2*, *HMGCR*, and *NPC1* did not change in the PDGF-BB treated HSCs (Figure 1D, right).

### 2.2. NPC2 Downregulation and Free Cholesterol Accumulation Enhanced PDGF-BB-Induced MAPK and AKT Activation in HSCs

**Figure 2:**
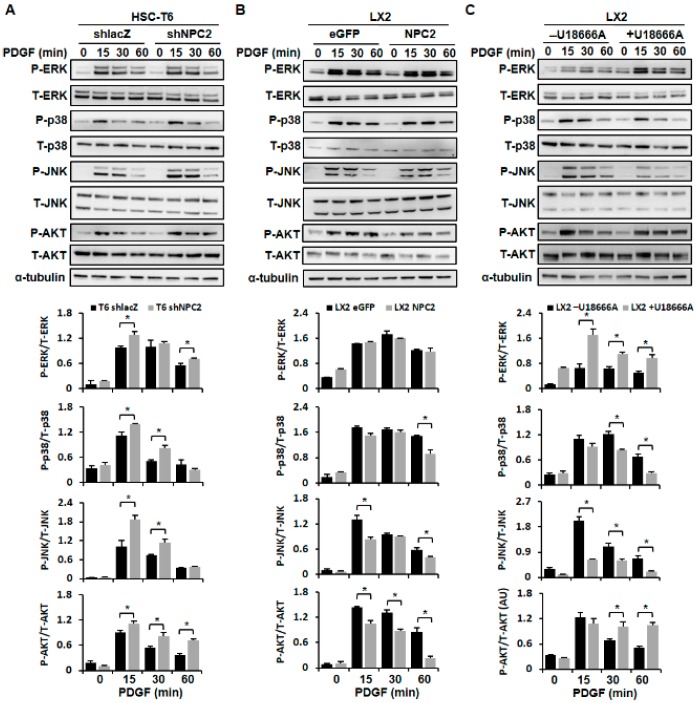
NPC2 downregulation and free cholesterol accumulation in the HSC-enhanced PDGF-BB-initiated MAPK and AKT signaling pathway activation. (**A**) shlacZ and shNPC2 HSC-T6 stable cells; (**B**) eGFP and NPC2 LX2 stable cells were treated with 10 ng/mL PDGF-BB for 0, 15, 30, and 60 minutes, and the cell lysates were subjected to Western blot analysis to detect ERK, p38, JNK, and AKT phosphorylation; (**C**) LX2 cells were pretreated with or without 1 µM U18666A for 24 h and added to 10 ng/mL PDGF-BB for the indicated time periods, and the lysates were immunoblotted to detect ERK, p38, JNK, and AKT phosphorylation. Each experiment was performed using three independent replicates with similar results, and representative data are shown in the figure. The quantification of the figure was presented below by using the ImageJ system. Data are shown as mean ± SD. * *p* < 0.05 vs. black bar.

It has been reported that PDGF-BB can stimulate cell proliferation by phosphorylating the MAPK and AKT pathways [8]. So we next tested whether NPC2 expression was involved in the PDGF-BB-initiated MAPK and AKT activation. As expected, downregulation of NPC2 in HSC-T6 cells resulted in enhancing PDGF-BB-induced ERK, p38, JNK, and AKT phosphorylation (Figure 2A); and upregulation of NPC2 in the LX2 cells attenuated PDGF-BB-induced phosphorylation of p38, JNK, and AKT, while the phosphor-ERK was nearly unchanged (Figure 2B). To determine the effect of U18666A on HSCs, the molecular mechanism of MAPK and AKT signaling activation was also studied. The data showed that free cholesterol that accumulated in the HSCs significantly increased the phosphorylation of ERK and AKT, while p38 and JNK phosphorylation was reduced (Figure 2C). Considered together, these results suggest that NPC2 expression is involved in the regulation of PDGF-BB-induced HSC proliferation through the MAPK and AKT pathways.

### 2.3. NPC2 Expression and Free Cholesterol Accumulation Did Not Alter HSC Apoptosis

**Figure 3:**
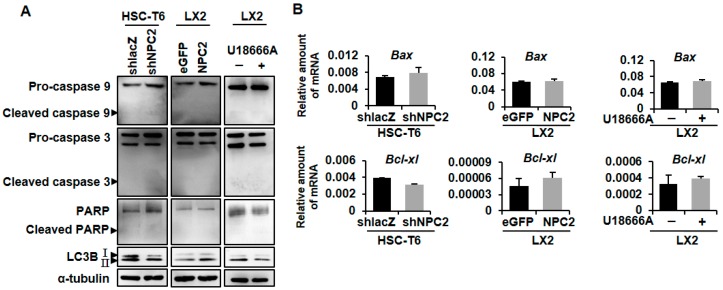
NPC2 expression and free cholesterol accumulation were not involved in the regulation of HSC apoptosis. (**A**) Protein expression of cleaved caspase 9, full caspase 9, cleaved caspase 3, full caspase 3, cleaved PARP, full PARP, LC3B, and α-tubulin was analyzed by using immunoblotting in NPC2-downregulated HSC-T6, NPC2-overexpressed LX2 and U18666A-treated LX2 cells; (**B**) Gene expression of pro-apoptotic *Bax* and anti-apoptotic *Bcl-xl* was analyzed by using real-time PCR in NPC2-downregulated HSC-T6 cells, NPC2-overexpressed LX2 cells, and U18666A-treated LX2 cells. Data are shown as mean ± SD. Each experiment was performed using three independent replicates. A similar phenomenon was observed, and representative data are shown in the figure.

Next, we examined the influence of NPC2 expression on HSC apoptosis. Western blot analyses for cleaved caspase 9, caspase 3, and poly (ADP-ribose) polymerase (PARP) were performed. The results showed that cleaved caspase 9, caspase 3, and PARP were barely detectable in both NPC2-downregulated and -overexpressed cells, while specific bands corresponding to full-length proteins were clearly detected (Figure 3A). At the same time, cleavage of caspase 9, caspase 3, and PARP was also undetected in U18666A-treated LX2 cells (Figure 3A). We then confirmed the result by evaluating the mRNA levels of the pro-apoptotic and anti-apoptotic genes, *Bax* and *Bcl-xl*. The results revealed that the expression of *Bax* and *Bcl-xl* did not change significantly in HSC-T6 shNPC2 cells, LX2 NPC2 cells, and U18666A-treated LX2 cells when compared with the relative control (Figure 3B). These data suggested that NPC2 expression and free cholesterol accumulation were not involved in the caspase-related apoptosis in HSCs. There have been some reports that have demonstrated that autophagy regulates cell death [30]. Therefore, we examined the levels of LC3B in the three groups. Western blot showed that both the HSC-T6 shNPC2 cells and U18666A-treated LX2 cells decreased the expression level of LC3B-II, but increased in LX2 NPC2 cells (Figure 3A).

### 2.4. NPC2 Downregulated and Free Cholesterol Accumulated HSCs Disrupt Mitochondrial Function 

It is known that mitochondria play a key role in cell metabolism [31], and these organelles are the key mechanism that controls cell growth and death [32]. Moreover, autophagy is the major clearance mechanism for eliminating dysfunctional mitochondria [33]. It has been reported that dysfunction of the autophagy pathway would cause the accumulation of dysfunctional mitochondria [34]. Therefore, we next investigated the mitochondrial function by using the Seahorse XFe extracellular flux analyzer (Agilent Technologies, Santa Clara, CA, USA). The steady-state oxygen consumption rate (OCR) was measured, and then oligomycin (Oligo) was injected into the system to inhibit ATP synthase. carbonyl cyanide 4-(trifluoromethoxy)phenylhydrazone (FCCP) was later added to analyze the maximal oxygen consumption. Finally, a rotenone and antimycin A (AA) mixture was injected to shut down the respiratory electron-transport chain for measuring the non-mitochondrial respiration. 

**Figure 4:**
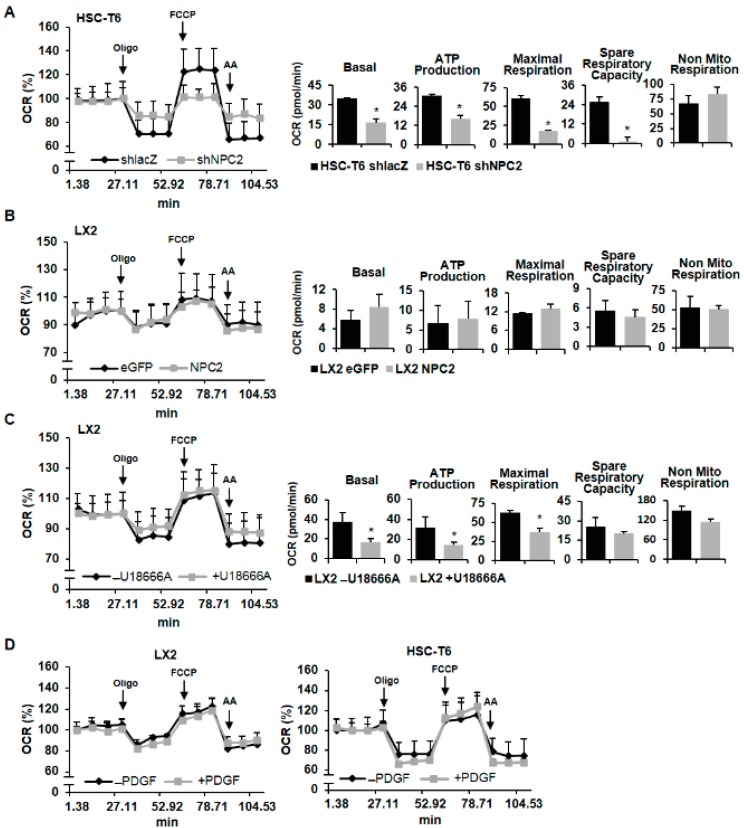
Downregulating NPC2 expression and accumulating free cholesterol in HSCs caused mitochondrial function disruption. (**A**,**B**) Mitochondrial respiration functions of NPC2-downregulated and -overexpressed cells were analyzed by a Seahorse XFe extracellular flux analyzer. A quantity of 1 μM oligomycin, 2 μM FCCP, and 0.5 μM rotenene/antimycin A was injected into the well at the 4th, 8th, and 11th time point. Quantitative data are shown in the right panel. (**C**) LX2 cells were pretreated with or without 1 µM U18666A for 24 h, and the oxygen consumption rate was analyzed by a Seahorse XFe extracellular flux analyzer. (**D**) LX2 and HSC-T6 cells were pretreated with or without 10 ng/mL PDGF-BB overnight and then subjected to the Seahorse XFe extracellular flux analyzer to analyze the mitochondrial respiration functions. Data are shown as mean ± SD. * *p* < 0.05 vs. black bar.

Figure 4A shows that the basal respiration of mitochondria was decreased in NPC2 knockdown HSC-T6 cells. In addition, HSC-T6 shNPC2 cells showed less oxygen participated in ATP production. Moreover, FCCP-induced maximal respiratory capacity was also decreased in NPC2-downregulated cells. Spare respiratory capacity was calculated by maximal respiration minus basal respiration, and it was also decreased in HSC-T6 shNPC2 cells, which indicates poor flexibility of mitochondria towards stress. Non-mitochondrial respiration was similar in both the shlacZ control and NPC2-downregulated cells. On the other hand, NPC2 overexpression cells had no significant difference of mitochondrial respiratory function and non-mitochondrial respiration when compared with eGFP control cells (Figure 4B). U18666A-induced free cholesterol accumulation in LX2 cells showed less oxygen consumption rate in basal respiration, ATP production, and maximal respiratory capacity, while spare respiratory capacity and non-mitochondrial respiration showed no statistical significance when compared to the control (Figure 4C). Next, we further examined whether PDGF-BB influenced HSCs mitochondrial function. The results showed that PEGF-BB treatment did not alter the LX2 or HSC-T6 cells’ mitochondrial function (Figure 4D). Taken together, these results indicate that both NPC2-downregulation and free cholesterol accumulation in the HSCs disrupted the mitochondria’s function. 

### 2.5. NPC2 Expression Was Not Involved in LPS-Induced Inflammatory Response, While U18666A-Treated HSCs Enhanced LPS-Induced Inflammation

**Figure 5:**
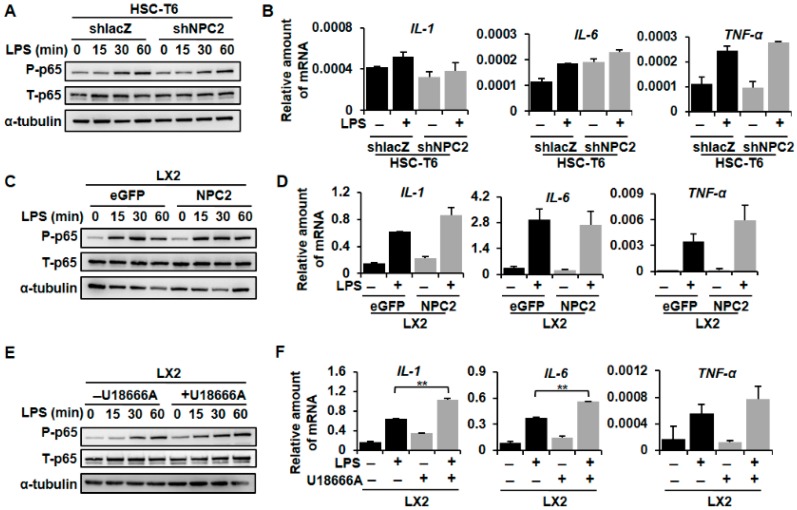

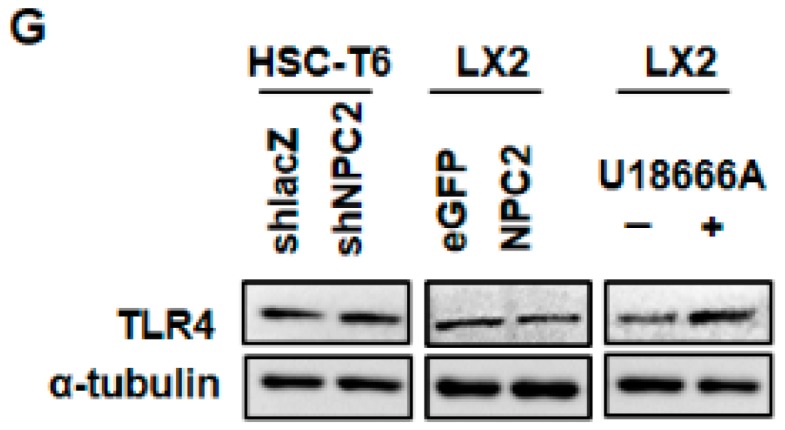
NPC2 expression did not alter the LPS-induced inflammatory response, while U18666A-treated HSCs amplified LPS-induced inflammation. (**A**) HSC-T6 shlacZ and shNPC2 stable cells were treated with 100 ng/mL LPS for the indicated time periods, and the lysates were subjected to Western blot analysis and detect p65 phosphorylation; (**B**) HSC-T6 shlacZ and shNPC2 cells were pretreated with 100 ng/mL LPS for 3 h, and mRNA expression of *IL-1*, *IL-6*, and *TNF-α* was analyzed using real-time PCR; (**C**) LX2 eGFP and NPC2 stable cells were treated with 10 ng/mL LPS for the indicated time periods, and the lysates were immunoblotted and then quantified to detect p65 phosphorylation; (**D**) Real-Time PCR was used to analyze mRNA expression of LPS-induced IL-1, IL-6, and TNF-α. (**E**,**F**) LX2 cells were pretreated with or without 1 µM U18666A overnight to induce free cholesterol accumulation and then treated with 100 ng/mL LPS. Cell lysates were immunoblotted to detect p65 phosphorylation, while real-time PCR was used to analyze mRNA expression of LPS-induced *IL-1*, *IL-6*, and *TNF-α*; (**G**) Protein expression of TLR4 and α-tubulin was analyzed by using immunoblotting in NPC2-downregulated HSC-T6, NPC2-overexpressed LX2, and U18666A-treated LX2 cells. Data are shown as mean ± SD. ** *p* < 0.01 vs. black bar. Each experiment was performed using three independent replicates. A similar phenomenon was observed. Therefore, representative data are shown in the figure.

It is widely believed that inflammation contributes to hepatic fibrosis in various types of liver diseases [14]. Moreover, the LPS/TLR4 signaling pathway in HSCs plays an important role in liver fibrogenesis [13]. To validate the functional role of NPC2 in inflammatory response, we tested whether NPC2 expression was involved in the LPS-induced p65 phosphorylation and downstream cytokine gene expression. Compared to the shlacZ control, the shNPC2 cells had no significant difference in phosphor-p65 expression after LPS treatment (Figure 5A). As expected, LPS-induced mRNA expression of *IL-1*, *IL-6*, and tumor necrosis factor (*TNF*)*-α* had no statistical significance between the shlacZ control and NPC2 knockdown cells (Figure 5B). On the other hand, the amount of LPS-induced p65 phosphorylation in NPC2-overexpressed LX2 cells was similar to the LX2 eGFP control cells (Figure 5C). At the same time, LPS-induced *IL-1*, *IL-6* and *TNF-α* mRNA expression also had no statistical significance between the eGFP control and NPC2 overexpression cells (Figure 5D). U18666A was used to induce free cholesterol accumulation in the LX2 cells. Importantly, the level of LPS-induced phosphor-p65 was significantly elevated after treatment with U18666A (Figure 5E). Consistent with this, *IL-1* and *IL-6* mRNA expression was increased in the U18666A-treated LX2 cells, while *TNF-α* mRNA expression had no statistical significance when compared to the control (Figure 5F). Since TLR4 is the receptor of LPS [13], we used Western blot to analyze the protein expression of TLR4 in the three different groups. Interestingly, U18666A-treated LX2 cells showed a significant increase in the TLR4 protein while the HSC-T6 shNPC2 cells and LX2 NPC2 cells remained unchanged (Figure 5G). Taken together, these results suggest that the expression of NPC2 did not alter TLR4 expression or influence LPS-induced inflammatory signals in HSCs, while U18666A-induced free cholesterol accumulation will sensitize HSCs toward LPS treatment through increased TLR4 expression.

## 3. Discussion

**Figure 6:**
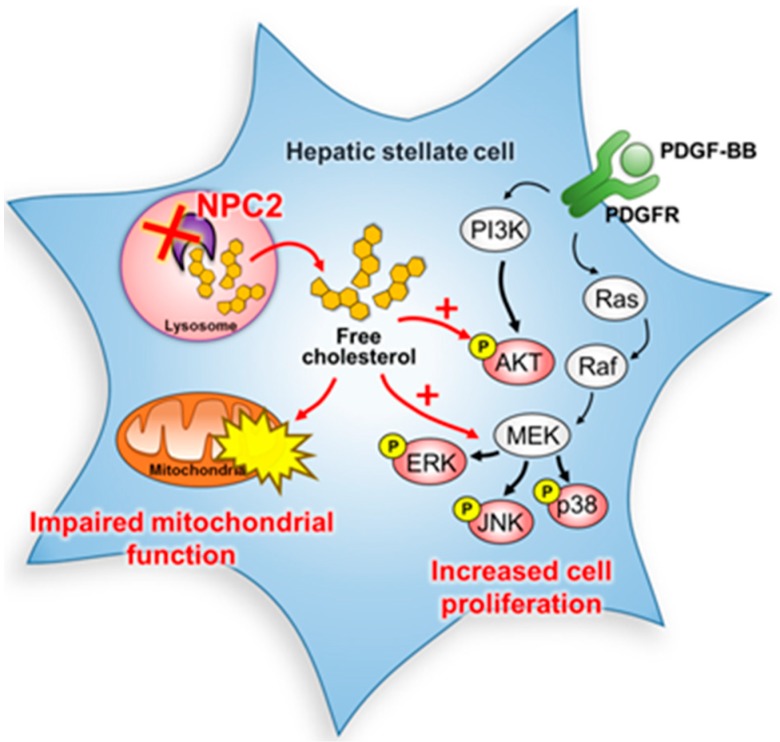
Schematic diagram of the signaling pathways involved in the downregulation of NPC2 in HSCs. Knockdown NPC2 in HSCs resulted in free cholesterol accumulation and enhanced the PDGF-BB-induced HSC proliferation by increasing the downstream proteins of MAPK and AKT phosphorylation. In addition, the mitochondrial respiration function was also impaired. ×, NPC2 downregulation. Black arrows, pathway signals. Red arrows and plus sign, proposed signals.

NPC2 was first characterized as a major secretory protein in the human epididymis to function as a key regulator in free cholesterol homeostasis [20,35]. We previously provided evidence that the downregulation of NPC2 is correlated with the α-fetoprotein level, tumor type, vascular invasion, and pathology stages of hepatocellular carcinoma (HCC) patients. In vitro and in vivo xenograft data demonstrated that the expression of NPC2 regulates cell proliferation, migration, and tumorigenesis by regulating ERK1/2 activation [36]. However, the roles of NPC2 in liver fibrosis have not been explored in detail. In 2016, we reported that NPC2 is decreased in both thioacetimidic acid (TAA)- and CCl_4_-induced mouse liver fibrosis tissues. Knockdown of NPC2 in HSCs markedly increased TGF-β1-induced collagen type 1 alpha 1, α-SMA expression, and smad2/3 phosphorylation. In contrast, NPC2 overexpression decreased TGF-β1-induced HSCs activation [24]. In this study, we showed that knockdown NPC2 and free cholesterol accumulation in HSCs could lead to the sensitization of HSCs to PDGF-BB induced proliferation by enhancing the phosphorylation of ERK, p38, JNK, and AKT (Figure 6). In addition, a lower oxygen consumption rate in basal mitochondria respiration, ATP production, and maximal respiratory capacity were also observed in NPC2 knockdown and free cholesterol accumulated HSCs (Figure 6). Our results support novel roles of NPC2 deficiency-mediated free cholesterol accumulation in the regulation of HSC proliferation and mitochondrial respiration function. 

Proliferation is the first perpetuation stage during HSCs activation [37]. PDGF-BB, a strong HSC mitogen, activates the Ras/ERK, JNK1/2, and PI3K/AKT signaling pathways [38]. In this study, we showed that NPC2 downregulation-induced free cholesterol accumulation promoted PDFG-BB-induced HSC proliferation (Figure 1). Our previous study demonstrated that NPC2 downregulation enhanced the proliferation of hepatocytes via the activation of ERK1/2. However, U18666A treatment-induced free cholesterol overload did not alter the proliferation and MAPK/ERK activation in hepatocytes [36]. Accordingly, these results suggest that HSCs, rather than hepatocytes, may serve as a major cause of HSCs proliferation resulting from NPC2 downregulation-dependent free cholesterol accumulation.

The role of apoptosis demonstrates an opposite role to cell proliferation in the regulation of cell populations [39]. The activation of caspases can activate other pro-caspases such as caspase 9, caspase 3, and PARP, allowing the initiation and amplification of the apoptotic signaling pathway and thus leading to rapid cell death [40]. However, our study showed that NPC2 expression was not involved in the caspase-related apoptosis in HSCs (Figure 3). Autophagy is another process associated with cell death by controlling cell degradation and metabolism of its own cytoplasm and organelles [41]. Microtubule-associated protein light chain 3β (LC3B) is a regulatory protein that is present from the autophagosome formation to the fusion with lysosomes [42]. In our study, we found that NPC2 knockdown HSCs decreased LC3B expression (Figure 3), which may demonstrate the reduction of autophagy activity. Shimizu et al. found that simian virus 40-transformed embryonic fibroblasts from *Bax/Bak* double knockout mice (B*ak*^+/−^ mice were crossed with *Bax*^+/−^ mice) underwent a non-apoptotic death after etoposide treatment, while the phenomenon was suppressed by inhibiting autophagy [43]. Moreover, lipid accumulation in human liver could cause a decrease in autophagic function [44]. Therefore, our study provided evidence that NPC2-knockdown caused free cholesterol accumulation that may impair the autophagic function, thus inhibiting autophagy-related cell death.

Mitochondria play a key role in cell metabolism [31], which is the key mechanism controlling cell growth and death [32]. Besides, mitochondria are the primary site for β-oxidation, where fatty acids are broken down, and produce energy during nutrient stress [45]. Accumulating evidence indicates that mitochondrial dysfunction is associated with abnormalities in lipid metabolism [46,47]. For instance, mitochondrial dysfunction could cause intracellular fat accumulation in NASH patients [46]. Moreover, alterations of lysosomal-mitochondrial liaisons via accumulating free cholesterol may result in mitochondrial dysfunction and defective antioxidant defense, which contribute to NPC disease progression [48,49]. A previous study showed that NPC2 deficiency led to impaired autophagic flux and decreased induction of LC3B during mitochondrial stress in adipocytes [50]. In this study, we found that NPC2 deficiency and U18666A-induced free cholesterol overload in HSCs impaired mitochondrial respiration function (Figure 4) without affecting apoptosis (Figure 3). Since autophagy-lysosomal mediated breakdown of intracellular lipid droplets provides a key source of energy for fueling HSC activation [51,52], NPC2-mediated free cholesterol homeostasis may play a critical role in regulating HSC energy metabolism during HSC activation and liver fibrosis. Moreover, it is well known that the autophagy pathway recognizes damaged mitochondria and triggers their engulfment [53]; therefore, dysfunction of the autophagy pathway may cause the accumulation of dysfunctional mitochondria [34]. Our result demonstrated that NPC2 knockdown HSCs exhibit decreased induction of LC3B; it is possible that NPC2 deficiency may impair mitophagy and reduce the removal of damaged mitochondria, which leads to mitochondrial dysfunction. Although more results are needed to determine the exact role of NPC2 in affecting autophagy and mitochondrial function, our results indicate a similar function in HSCs as has been observed in other cell types. 

The persistent inflammatory response is considered as the major driving force sustaining fibrogenesis. Inflammation presents in most patients with hepatic fibrosis and correlates with fibrosis progression [2]. The intestine-derived bacterial product LPS may reach the liver through the portal vein and activate the hepatic immune system [54]. HSCs are activated when LPS binds to TLR4 which sensitizes HSCs to TGF-β-induced activation through the NF-κB (p65) pathway [55]. Tomita et al. showed that increased cholesterol intake in mice would accelerate liver fibrosis due to free cholesterol accumulation in the hepatic stellate cells and further increase TLR4 protein expression [56]. In our research, we found that U18666A treatment-induced HSC-free cholesterol accumulation enhanced LPS-induced NF-κB phosphorylation, IL-1, and IL-6 expression (Figure 5E,F). However, the expression of NPC2 did not alter LPS-induced inflammatory signals and NF-κB phosphorylation (Figure 5A–D). The protein level of TLR4 was also measured and the results showed that U18666A-treated LX2 had increased TLR4 expression while HSC-T6 shNPC2 and LX2 NPC2 cells had no significant change (Figure 5G). Our result is similar to that from a previous study, which showed that U18666A treatment would enhance NF-κB activity through the TLR4 signaling pathway [57]. These may give an explanation for the different inflammatory responses between NPC2 knockdown and U18666A-treated groups after LPS treatment. Besides, from our previous research, we found that knockdown NPC2 in HSC-T6 cells would increase 20% free cholesterol accumulation [24]. Since U18666A is a specific drug that induces free cholesterol sequestration in late endosomes/lysosomes and mimics an NPC phenotype [27,28,29], the efficiency of U18666A showed a more than 50% increase in cholesterol accumulation when compared to the control [26,58]. Together, these results suggest that increased TLR4 expression in free cholesterol accumulated HSCs may be a key factor in responding to LPS stimulation.

In summary, our study has provided new insights into the mechanisms linking NPC2 deficiency-induced free cholesterol accumulation, which influences HSC proliferation and mitochondrial function. Regulating NPC2 in HSCs may therefore represent a new treatment strategy for liver fibrosis.

## 4. Materials and Methods 

### 4.1. Cell Culture, Plasmids, and Lentiviral Infection

293T cells lines were purchased from ATCC (No. CRL-11268, Manassas, VA, USA) and were cultured in Dulbecco’s modified Eagle’s medium (DMEM; Gibco BRL, Grand Island, NY, USA) supplemented with 10% fetal bovine serum (HyClone, Logan, UT, USA), penicillin (10,000 Unit/mL) and streptomycin (10,000 μg/mL), nonessential amino acids (0.1 mM), and l-glutamine (2 mM) at 37 °C in a 5% CO_2_ incubator. Gene knockdown used RNA interference experiments and the plasmid encoded NPC2 shRNA (pLKO.1-shNPC2) and control lacZ shRNA (pLKO.1-shlacZ). For the overexpression experiments, the plasmids (pLKO_AS3w.eGFP.puro, and pLV-NPC2) were obtained from the National RNAi Core Facility (Academia Sinica, Taipei, Taiwan). We used TurboFectTM Reagent (Fermentas, Hanover, MD, USA) for the co-transfected packaging plasmid-pCMV-DR8.91, VSV-G envelope expressing plasmid-pMD.G, and one of four lentiviral constructs (pLKO.1-shlacZ, pLKO.1-shNPC2, pLKO_AS3w.eGFP.puro, pLV-NPC2) in 293T cells. Supernatants with lentiviruses were collected. To produce stable cell lines, LX2 cells and HSC-T6 cells were infected with lentivirus in polybrene (8 μg/mL) medium. After 24 h infection, 1 μg/mL purumycin was added for selecting stable cells. Gene-transfected stable cells (HSC-T6 shlacZ, HSC-T6 shNPC2, LX2 eGFP, and LX2 NPC2) were grown in DMEM with 1% fetal bovine serum (FBS) and 1 μg/mL puromycin [24].

### 4.2. AlamarBlue^®^ Cell Viability Assay

Cell growth was determined using the commercial alamarBlue^®^ cell viability reagent (Life Technologies, Carlsbad, CA, USA). Different gene-modified HSCs were seeded (2.5 × 10^3^) in a 96-well plate and treated with PDGF. After every 24 h treatment period, 10 mL of alamarBlue^®^ reagent was added to 100 mL culture media and incubated for 2.5 h at 37 °C in a 5% CO_2_ incubator. The nontoxic alamarBlue^®^ (resazurin) enters into the living cells and is reduced by mitochondrial FMNH2, FADH2, NADH, NADPH, and cytochromes. The ingredient transforms from blue non-fluorescent resazurin to pink fluorescent resorufin. The absorbance was evaluated at 570 nm, and we used 600 nm as the reference wavelength (normalized to the 600 nm value) to quantitatively measure the proliferation of cultured cells.

### 4.3. BrdU Assay

Different gene-modified HSCs were seeded (2.5 × 10^3^) in a 96-well plate and treated with PDGF-BB. After 72 h treatment periods, cells were incubated in a BrdU solution (BioVision, Milpitas, CA, USA) for 2.5 h, followed by incubation in a fixing/denaturing solution for 30 min. They were then reacted with a BrdU detection antibody for 1 h and an anti-mouse horseradish peroxidase (HRP)-linked antibody for 1 h. The level of cell proliferation was determined by measuring absorbance at 450 nm.

### 4.4. PDGF-BB, LPS, and U18666A Treatment

For studying the effect of NPC2 expression on PDGF-induced cell proliferation and LPS-induced cell inflammation, HSCs were seeded in 6 well plate (1.5 × 10^5^) and treated with 10 ng/mL PDGF (R&D Systems, Minneapolis, MN, USA) and 100 ng/mL LPS (Sigma-Aldrich, St Louis, MO, USA) for different time periods, respectively. HSCs were pretreated with 1 µM U18666A overnight to induce the accumulation of free cholesterol and then treated with PDGF-BB.

### 4.5. Western Blot

Total proteins were extracted from cultured cells using lysis buffer supplemented with protease and phosphatase inhibitors. The protein concentration was measured by protein assay (Bio-Rad Laboratories, Hercules, CA, USA) and all samples were normalized to 30 μg. Cellular proteins were separated by sodium dodecyl sulfate–polyacrylamide gel electrophoresis (SDS-PAGE) and subjected to protein transfer onto PVDF membrane. The membrane used primary and secondary antibodies to catch the target. The following antibodies used in this study were purchased from Cell Signaling (Beverly, MA, USA): phospho-, and total-ERK, JNK, p38, p65, caspase 9, caspase 3, PARP, and LC3B. NPC2 was purchased from Santa Cruz Biotechnology (Santa Cruz, CA, USA). The bands were visualized using ECL detection reagent (Millipore Corporation, Billerica, MA, USA) and the immunoblotting signals were quantified by densitometric scanning (ImageJ software 1.47v, National Institutes of Health, Bethesda, MD, USA).

### 4.6. Real-Time PCR

Total RNA was isolated from cultured cells using TRIzol Reagent (Ambion, Carlsbad, CA, USA), according to the manufacturer’s protocol. A SuperScript II RNase H-Reverse Transcriptase Kit (Invitrogen, Carlsbad, CA, USA) was used to produce complementary DNA from cellular RNA (2 μg). The primers used in the real-time PCR are listed in Table 1. We added 4 µL template cDNA (20 ng), 5 µL KAPA SYBR^®^ FAST qPCR Master Mix (2×), and 1 µL forward/reverse primer mix (6 µM each) (KAPA Biosystems, Boston, MA, USA) into 48-well PCR plates for reactions (10 μL). Thermal cycling consisted of 15 min at 95 °C, followed by 40 cycles at 95 °C for 15 s and 60 °C for 60 s using the StepOne System (Applied Biosystems, Foster City, CA, USA). The predicted cycle threshold (C*_t_*) values were exported to Excel worksheets for analysis. Comparative C*_t_* methods, which were normalized to *GAPDH*, were used to determine the gene expression levels.

### 4.7. Free Cholesterol Quantification

The intracellular concentration of free cholesterol was measured using a commercial colorimetric kit (BioVision, Milpitas, CA, USA). 

### 4.8. Seahorse Assay

The in vivo cell real-time cellular oxygen consumption rate (OCR) was measured by an XF24 bioenergetic assay (Seahorse Bioscience, Billerica, MA, USA), according to the manufacturer’s instructions. Briefly, cells were seeded in a XF24-well plate containing complete medium. After 16 h, the XF24 bioenergetic assay was initiated by removing the exhausted medium and replacing it with sodium-bicarbonate-free DMEM containing 2% FBS. The OCR was detected at a steady state, then oligomycin (1 µM), carbonyl cyanide 4-(trifluoromethoxy)phenylhydrazone (FCCP; 2 μM), rotenone/antimycin A (AA; 0.5 μM) were injected at the 4th, 8th, and 11th time point into the wells to obtain the values of the maximal and non-mitochondrial respiration rate.

### 4.9. Statistical Analysis

Results are expressed as the mean ± standard deviation (SD). The data were analyzed by non-parametric tests using SPSS v20.0 (SPSS Inc, Chicago, IL, USA) software. The Mann–Whitney *U* test was used to compare two independent groups. Kruskal-Wallis followed by Bonferroni *post hoc* analyses was used to account for multiple testing. Differences were considered statistically significant at *p* < 0.05.

## Figures and Tables

**Table 1:** Primers used for real-time PCR.

Gene	Forward Primer (5′→3′)	Reverse Primer (3′→5′)
*GAPDH*	TCACCACCATGGAGAAGGC	GCTAAGCAGTTGGTGGTGCA
*NPC2*	CGGAGCCCCTGCACTTC	GGGCTCACATTCACCTCCTTTA
*Bax*	GATCCAGGATCGAGCAGA	AAGTAGAAGAGGGCAACCAC
*Bcl-xl*	GCTGCATTGTTCCCGTAGAG	GTTGGATGGCCACCTATCTG
*Abca1*	CCCCTGCTTCCGTTATCCA	GGACCTTGTGCATGTCCTTAATG
*Abcb11*	CAGAACATGACAAACGGAACAAG	CCTGCGTAGATGCCAGAAAATT
*Cyp7A1*	ACTCTCTGAAGCCATGATGCAA	ACCCAGACAGCGCTCTTTGA
*SREBP2*	GCTACCGGTCCTCCATCAAC	ACGCCAGACTTGTGCATCTTG
*HMGCR*	TGTGGTTTGTGAAGCCGTCAT	TCAACCATAGCTTCCGTAGTTGTC
*NPC1*	AACAAAGTAGACATCGGGTTGGA	ACTGAGCGAGTGATTTGAAATAGTCA
h-*IL-1*	CTGAGCACCTTCTTTCCCTTCA	TGGACCAGACATCACCAAGCT
h-*IL-6*	TGGCTGAAAAAGATGGATGCT	TCTGCACAGCTCTGGCTTGT
h-*TNF-α*	TGTAGCCCATGTTGTAGCAAACC	GAGGACCTGGGAGTAGATGAGGTA
m-*IL-1*	TCCAGGATGAGGACATGAGCAC	GAACGTCACACACCAGCAGGTTA
m-*IL-6*	GGACTGATGCTGGTGAC	CATTTCTTTGTATCTCTGGAAGTT
m-*TNF-α*	CAAGACTGACAACCAGACG	ACAGAAGCAGAGATTATGACC

## Abbreviations

HSCs	Hepatic stellate cells
NPC2	Niemann-Pick type C2
PDGF-BB	Platelet-derived growth factor BB
MAPK/ERK	Mitogen-activated protein kinases
α-SMA	α-Smooth muscle actin
LPS	Lipopolysaccharide
TLR4	Toll-like receptor 4
OCR	Oxygen consumption rate
Oligo	Oligomycin
AA	Rotenone and antimycin A

## References

[1] Pellicoro A., Ramachandran P., Iredale J.P., Fallowfield J.A. (2014). Liver fibrosis and repair: Immune regulation of wound healing in a solid organ. Nat. Rev. Immunol..

[2] Bataller R., Brenner D.A. (2005). Liver fibrosis. J. Clin. Invest..

[3] Geerts A. (2001). History, heterogeneity, developmental biology, and functions of quiescent hepatic stellate cells. Semin. Liver Dis..

[4] Carson J.P., Ramm G.A., Robinson M.W., McManus D.P., Gobert G.N. (2018). Schistosome-induced fibrotic disease: The role of hepatic stellate cells. Trends Parasitol..

[5] Moreira R.K. (2007). Hepatic stellate cells and liver fibrosis. Arch. Pathol. Lab. Med..

[6] Lee U.E., Friedman S.L. (2011). Mechanisms of hepatic fibrogenesis. Best Pract. Res. Clin. Gastroenterol..

[7] Zhang D.Y., Friedman S.L. (2012). Fibrosis-dependent mechanisms of hepatocarcinogenesis. Hepatology.

[8] Borkham-Kamphorst E., Weiskirchen R. (2016). The PDGF system and its antagonists in liver fibrosis. Cytokine Growth Factor Rev..

[9] Van Dijk F., Olinga P., Poelstra K., Beljaars L. (2015). Targeted therapies in liver fibrosis: Combining the best parts of platelet-derived growth factor BB and interferon gamma. Front. Med..

[10] Pinzani M., Milani S., Herbst H., DeFranco R., Grappone C., Gentilini A., Caligiuri A., Pellegrini G., Ngo D.V., Romanelli R.G. (1996). Expression of platelet-derived growth factor and its receptors in normal human liver and during active hepatic fibrogenesis. Am. J. Pathol..

[11] Shi H., Dong L., Dang X., Liu Y., Jiang J., Wang Y., Lu X., Guo X. (2013). Effect of chlorogenic acid on LPS-induced proinflammatory signaling in hepatic stellate cells. Inflamm. Res..

[12] Seki E., Schwabe R.F. (2015). Hepatic inflammation and fibrosis: Functional links and key pathways. Hepatology.

[13] Paik Y.H., Schwabe R.F., Bataller R., Russo M.P., Jobin C., Brenner D.A. (2003). Toll-like receptor 4 mediates inflammatory signaling by bacterial lipopolysaccharide in human hepatic stellate cells. Hepatology.

[14] Schwabe R.F., Seki E., Brenner D.A. (2006). Toll-like receptor signaling in the liver. Gastroenterology.

[15] Akira S., Uematsu S., Takeuchi O. (2006). Pathogen recognition and innate immunity. Cell.

[16] Ioannou G.N., Morrow O.B., Connole M.L., Lee S.P. (2009). Association between dietary nutrient composition and the incidence of cirrhosis or liver cancer in the united states population. Hepatology.

[17] Teratani T., Tomita K., Suzuki T., Oshikawa T., Yokoyama H., Shimamura K., Tominaga S., Hiroi S., Irie R., Okada Y. (2012). A high-cholesterol diet exacerbates liver fibrosis in mice via accumulation of free cholesterol in hepatic stellate cells. Gastroenterology.

[18] Tomita K., Teratani T., Suzuki T., Shimizu M., Sato H., Narimatsu K., Usui S., Furuhashi H., Kimura A., Nishiyama K. (2014). Acyl-CoA:Cholesterol acyltransferase 1 mediates liver fibrosis by regulating free cholesterol accumulation in hepatic stellate cells. J. Hepatol..

[19] Ko D.C., Binkley J., Sidow A., Scott M.P. (2003). The integrity of a cholesterol-binding pocket in Niemann-Pick C2 protein is necessary to control lysosome cholesterol levels. Proc. Natl. Acad. Sci. USA.

[20] Storch J., Xu Z. (2009). Niemann-Pick C2 (NPC2) and intracellular cholesterol trafficking. Biochim. Biophys. Acta.

[21] Reif S., Spirer Z., Messer G., Baratz M., Bembi B., Bujanover Y. (1994). Severe failure to thrive and liver dysfunction as the main manifestations of a new variant of Niemann-Pick disease. Clin. Pediatr..

[22] Kelly D.A., Portmann B., Mowat A.P., Sherlock S., Lake B.D. (1993). Niemann-Pick disease type C: Diagnosis and outcome in children, with particular reference to liver disease. J. Pediatr..

[23] Liao Y.J., Lin M.W., Yen C.H., Lin Y.T., Wang C.K., Huang S.F., Chen K.H., Yang C.P., Chen T.L., Hou M.F. (2013). Characterization of Niemann-Pick type C2 protein expression in multiple cancers using a novel NPC2 monoclonal antibody. PLoS ONE.

[24] Twu Y.C., Lee T.S., Lin Y.L., Hsu S.M., Wang Y.H., Liao C.Y., Wang C.K., Liang Y.C., Liao Y.J. (2016). Niemann-Pick type C2 protein mediates hepatic stellate cells activation by regulating free cholesterol accumulation. Int. J. Mol. Sci..

[25] Wong L., Yamasaki G., Johnson R.J., Friedman S.L. (1994). Induction of β-platelet-derived growth factor receptor in rat hepatic lipocytes during cellular activation in vivo and in culture. J. Clin. Invest..

[26] Copetti-Santos D., Moraes V., Weiler D.F., de Mello A.S., Machado Fde S., Marinho J.P., Siebert C., Kolling J., Funchal C., Wyse A.T. (2015). U18666a treatment results in cholesterol accumulation, reduced Na^+^, K^+^-ATPase activity, and increased oxidative stress in rat cortical astrocytes. Lipids.

[27] Liscum L., Faust J.R. (1989). The intracellular transport of low density lipoprotein-derived cholesterol is inhibited in chinese hamster ovary cells cultured with 3-β-[2-(diethylamino)ethoxy]androst-5-en-17-one. J. Biol. Chem..

[28] Liscum L., Munn N.J. (1999). Intracellular cholesterol transport. Biochim. Biophys. Acta.

[29] Ko D.C., Gordon M.D., Jin J.Y., Scott M.P. (2001). Dynamic movements of organelles containing Niemann-Pick C1 protein: NPC1 involvement in late endocytic events. Mol. Biol. Cell.

[30] Levine B., Yuan J. (2005). Autophagy in cell death: An innocent convict?. J. Clin. Investig..

[31] Cogliati S., Enriquez J.A., Scorrano L. (2016). Mitochondrial cristae: Where beauty meets functionality. Trends Biochem. Sci..

[32] Mason E.F., Rathmell J.C. (2011). Cell metabolism: An essential link between cell growth and apoptosis. Biochim. Biophys. Acta.

[33] Ashrafi G., Schwarz T.L. (2013). The pathways of mitophagy for quality control and clearance of mitochondria. Cell Death Differ..

[34] Luo C., Li Y., Wang H., Feng Z., Li Y., Long J., Liu J. (2013). Mitochondrial accumulation under oxidative stress is due to defects in autophagy. J. Cell. Biochem..

[35] Kirchhoff C., Osterhoff C., Young L. (1996). Molecular cloning and characterization of he1, a major secretory protein of the human epididymis. Biol. Reprod..

[36] Liao Y.J., Fang C.C., Yen C.H., Hsu S.M., Wang C.K., Huang S.F., Liang Y.C., Lin Y.Y., Chu Y.T., Arthur Chen Y.M. (2015). Niemann-Pick type C2 protein regulates liver cancer progression via modulating ERK1/2 pathway: Clinicopathological correlations and therapeutical implications. Int. J. Cancer.

[37] Friedman S.L. (2008). Hepatic fibrosis—Overview. Toxicology.

[38] Forbes S.J., Parola M. (2011). Liver fibrogenic cells. Best Pract. Res. Clin. Gastroenterol..

[39] Elmore S. (2007). Apoptosis: A review of programmed cell death. Toxicol. Pathol..

[40] Cohen G.M. (1997). Caspases: The executioners of apoptosis. Biochem. J..

[41] Glick D., Barth S., Macleod K.F. (2010). Autophagy: Cellular and molecular mechanisms. J. Pathol..

[42] Mizushima N., Yoshimori T., Levine B. (2010). Methods in mammalian autophagy research. Cell.

[43] Shimizu S., Kanaseki T., Mizushima N., Mizuta T., Arakawa-Kobayashi S., Thompson C.B., Tsujimoto Y. (2004). Role of BCL-2 family proteins in a non-apoptotic programmed cell death dependent on autophagy genes. Nat. Cell Biol..

[44] Cuervo A.M., Bergamini E., Brunk U.T., Droge W., Ffrench M., Terman A. (2005). Autophagy and aging: The importance of maintaining “clean” cells. Autophagy.

[45] Eaton S., Bartlett K., Pourfarzam M. (1996). Mammalian mitochondrial β-oxidation. Biochem. J..

[46] Pessayre D., Fromenty B. (2005). Nash: A mitochondrial disease. J. Hepatol..

[47] Perez-Carreras M., Del Hoyo P., Martin M.A., Rubio J.C., Martin A., Castellano G., Colina F., Arenas J., Solis-Herruzo J.A. (2003). Defective hepatic mitochondrial respiratory chain in patients with nonalcoholic steatohepatitis. Hepatology.

[48] Wos M., Szczepanowska J., Pikula S., Tylki-Szymanska A., Zablocki K., Bandorowicz-Pikula J. (2016). Mitochondrial dysfunction in fibroblasts derived from patients with Niemann-Pick type C disease. Arch. Biochem. Biophys..

[49] Torres S., Balboa E., Zanlungo S., Enrich C., Garcia-Ruiz C., Fernandez-Checa J.C. (2017). Lysosomal and mitochondrial liaisons in Niemann-Pick disease. Front. Physiol..

[50] Guo H., Zhao M., Qiu X., Deis J.A., Huang H., Tang Q.Q., Chen X. (2016). Niemann-Pick type C2 deficiency impairs autophagy-lysosomal activity, mitochondrial function, and TLR signaling in adipocytes. J. Lipid Res..

[51] Hernandez-Gea V., Ghiassi-Nejad Z., Rozenfeld R., Gordon R., Fiel M.I., Yue Z., Czaja M.J., Friedman S.L. (2012). Autophagy releases lipid that promotes fibrogenesis by activated hepatic stellate cells in mice and in human tissues. Gastroenterology.

[52] Thoen L.F., Guimaraes E.L., Dolle L., Mannaerts I., Najimi M., Sokal E., van Grunsven L.A. (2011). A role for autophagy during hepatic stellate cell activation. J. Hepatol..

[53] Elmore S.P., Qian T., Grissom S.F., Lemasters J.J. (2001). The mitochondrial permeability transition initiates autophagy in rat hepatocytes. FASEB J..

[54] Takeuchi O., Akira S. (2010). Pattern recognition receptors and inflammation. Cell.

[55] Tsuchida T., Friedman S.L. (2017). Mechanisms of hepatic stellate cell activation. Nat. Rev. Gastroenterol. Hepatol..

[56] Tomita K., Teratani T., Suzuki T., Shimizu M., Sato H., Narimatsu K., Okada Y., Kurihara C., Irie R., Yokoyama H. (2014). Free cholesterol accumulation in hepatic stellate cells: Mechanism of liver fibrosis aggravation in nonalcoholic steatohepatitis in mice. Hepatology.

[57] Suzuki M., Sugimoto Y., Ohsaki Y., Ueno M., Kato S., Kitamura Y., Hosokawa H., Davies J.P., Ioannou Y.A., Vanier M.T. (2007). Endosomal accumulation of toll-like receptor 4 causes constitutive secretion of cytokines and activation of signal transducers and activators of transcription in Niemann-Pick disease type C (NPC) fibroblasts: A potential basis for glial cell activation in the npc brain. J. Neurosci..

[58] Appelqvist H., Nilsson C., Garner B., Brown A.J., Kagedal K., Ollinger K. (2011). Attenuation of the lysosomal death pathway by lysosomal cholesterol accumulation. Am. J. Pathol..

